# Mobility and living status at discharge and after three-months for extramedullary versus intramedullary fixation of AO type 31-A1 trochanteric fractures; an analysis of Dutch hip fracture audit data

**DOI:** 10.1007/s00068-024-02749-8

**Published:** 2025-01-10

**Authors:** Miliaan L. Zeelenberg, Esther M. M. Van Lieshout, Taco Gosens, Johannes H. Hegeman, Dennis Den Hartog, Michael H. J. Verhofstad, Stable-HIP group, Pieter Joosse, Rudolf W. Poolman, Hanna C. Willems, Rutger G. Zuurmond, Dutch Hip Fracture Audit indicator Taskforce, G. De Klerk, O. C. Geraghty, H. A. F. Luning, A. H. P. Niggebrugge, M. Regtuijt, J. Snoek, C. Stevens, D. Van der Velde, E. J. M. M. Verleisdonk

**Affiliations:** 1 Trauma Research Unit Department of Surgery, Erasmus MC, University Medical Center Rotterdam, P.O. Box 2040, 3000 CA Rotterdam, The Netherlands; 2 Department of Orthopedics, Elisabeth Hospital (ETZ), Tilburg, The Netherlands; 3 Department of Trauma Surgery, Ziekenhuisgroep Twente, Almelo, The Netherlands

**Keywords:** Trochanteric fracture, AO type 31-A1, Intramedullary fixation, Extramedullary fixation, Dutch hip fracture audit

## Abstract

**Purpose:**

The use of intramedullary fixation of AO type 31-A1 fractures is rising, despite evidence of non-superiority when compared with extramedullary fixation. The aim of this study was to evaluate mobility and living status for extramedullary fixation (EMF) versus intramedullary fixation (IMF) in Dutch hospitals during the initial hospital stay and until three-months after trauma.

**Methods:**

Data on patient characteristics, mobility, living status, complications, reoperation, and mortality were extracted from the Dutch Hip Fracture Audit Indicator Taskforce. Data were collected for patients (> 65 years) at baseline, at discharge, and at three-months follow-up. Univariate analysis was used for comparing the EMF versus IMF group.

**Results:**

A total of 836 patients were included; 138 (16%) were treated with EMF and 698 (84%) with IMF. No significant differences were found between groups for the overall complication rate during the initial hospital stay (EMF: n = 55 (40%) versus IMF: n = 270 (39%)). Patients treated with EMF showed better mobility at discharge (mobility with frame/2 supports or better, EMF 77% versus IMF 50%), but otherwise no significant difference was found after a three-month follow-up (EMF 80% versus IMF 82%), suggesting faster improved mobility for IMF. However, matched subgroup analysis showed no meaningful differences in rates of deteriorated mobility or living status after three months.

**Conclusion:**

This study showed no meaningful differences between EMF and IMF of type 31-A1 trochanteric fractures during hospital stay and until three-month follow-up. Despite little differences in outcome and EMF being the treatment option of first choice by the Dutch Hip fracture guideline, IMF is used in the vast majority of patients.

**Supplementary Information:**

The online version contains supplementary material available at 10.1007/s00068-024-02749-8.

## Introduction

A hip fracture is one of the leading causes of mortality and disability in the elderly population worldwide [1, 2]. Current global incidence rate was estimated at 182.5 per 100,000 person-years, and these numbers are projected to increase even further in the upcoming decades [3]. Trochanteric fractures constitute roughly 40–50% of the total number of hip fractures [3, 4]. They were subdivided by the Arbeitsgemeinschaft für Osteosynthesefragen (AO) 2018 classification [5] into three main subcategories: 31-A1 simple (stable) pertrochanteric fractures; 31-A2 multi-fragmentary pertrochanteric, lateral wall incompetent (< 20.5 mm) fractures; and 31-A3 intertrochanteric (reverse obliquity) fractures. These fractures are usually fixated using either an extramedullary technique (i.e., a type of sliding hip screw) or an intramedullary technique (i.e., a type of intramedullary nail). The ideal fixation technique varies per type of fracture. While the American Academy of Orthopaedic Surgeons advises the usage of both intramedullary and extramedullary devices for A1 trochanteric fractures, several international guidelines advise extramedullary fixation for A1 fractures and consider both extra- and intramedullary fixation as a viable option in A2 fractures [6–8].

Despite these guidelines, the use of intramedullary fixation in all fracture types, and specifically A1 fractures, has risen in the past decades [9–12]. Recent studies show increasing effectiveness of intramedullary devices but superiority in A1 fractures remains questionable [13–15]. Factors that are attributed to their increased usage are surgeons’ training, treatment volume of hospitals, and geographical differences, and the change does not seem to correlate with only patient-specific differences [11, 16]. The Dutch hip fracture guideline also advises extramedullary fixation for the stable A1 subgroup due to lack of superiority for either treatment approach and lower implant costs for extramedullary devices [7]. Despite this, but in line with previously mentioned international changes, a large majority of patients is treated using intramedullary devices [17]. Due to the evolution of implants or improved operation techniques, and decreased usage of extramedullary devices, the results of intramedullary fixation may have improved further. The question is whether this trend of increasing intramedullary nailing has persisted in stable trochanteric fractures and if patient outcomes and complication rates have remained similar or have surpassed the outcomes of extramedullary devices.

As no recent large-scale outcome comparison for both devices exists for the Dutch population, this study aimed to compare the use of extramedullary versus intramedullary fixation of stable trochanteric fractures (AO type 31-A1) in older adults and compare mobility and living status at discharge and three months after trauma.

## Methods

This retrospective cohort study was performed using data from the Dutch Hip Fracture Audit (DHFA) of the period January 1, 2018 to December 31, 2020 [18]. These data are collected annually by the Dutch Institute for Clinical Auditing (DICA) [19]. The DHFA has national coverage, and includes all hip fracture patients, but not all data, especially data regarding follow-up, is completed to a satisfactory level by the participating hospitals. Because of this, the DHFA Indicator Taskforce was created: a group consisting of six mid-to-large-sized Dutch hospitals (see list in acknowledgments) committed to adequate registration of treatment specific- and follow-up data. To ensure comparison of adequate data with a well completed follow-up this analysis was restricted to the data provided by the DHFA Indicator Taskforce.

Patients were included if they were ≥ 65 years and were admitted with an acute AO type 31-A1 trochanteric proximal femoral fracture. Data were collected on baseline characteristics (age, sex, ASA classification, pre-fracture mobility, pre-fracture Parker mobility score (PMS), pre-fracture Katz-ADL score, and pre-fracture living status), operation characteristics (implant type, time-to-surgery, type of anesthesia), hospital stay, complications as classified by hospital in DHFA, mobility at discharge, and discharge location. Additional data was collected after a three-month follow-up (reoperation, living status, mobility, Katz-ADL score, and mortality). Primary outcomes were (changes in) mobility and living status at discharge and at three-months after trauma. Secondary outcomes were mortality, in-hospital complications, HLOS, Katz-ADL at discharge and three months, and reoperation rate until three months.

Mobility was classified as either independent, mobility with 1 or 2 supports, mobility only within own home, or no functional mobility. Living status was classified as: at home with, or without supportive care, care home, nursing home, or rehabilitation institution. The latter three were seen as institutionalized and the first two as community-dwelling. The Katz-ADL score provides an indication of the ability to perform basic activities of daily life (ADL) [20]. A score of zero indicates independent function and six indicates total dependance in ADL tasks.

### Statistical analysis

Data were analyzed using the Statistical Package for the Social Sciences (SPSS) version 28.0 (SPSS, Chicago, Ill., USA). Normality for continuous data was assessed using the Shapiro–Wilk test. Missing data were not imputed. The analyses were done stratified for extramedullary fixation (EMF) versus intramedullary fixation (IMF). To correct for possible confounders in the main analysis a matched complete case analysis was performed for all three month outcomes. This included only patients with complete baseline characteristics and three-month outcomes. Cases (EMF) were matched 1:2 without replacement with controls (IMF), on age (within 10 years), sex, ASA classification, and pre-fracture mobility. In addition, a sensitivity analysis comparing EMF and IMF was performed for the total population, stratified on living status (institutionalized versus community dwelling).

Continuous data were reported as median with first quartile and third quartile and categorical data as numbers with percentages. Univariate comparison between groups in the unmatched analysis was performed using a Mann–Whitney or χ^2^ test. For the matched analysis, the Mantel–Haenszel statistic was used to calculate odds ratios for outcomes after three months. A 2-sided P-value < 0.05 was used as the threshold for statistical significance.

## Results

**Fig. 1:**
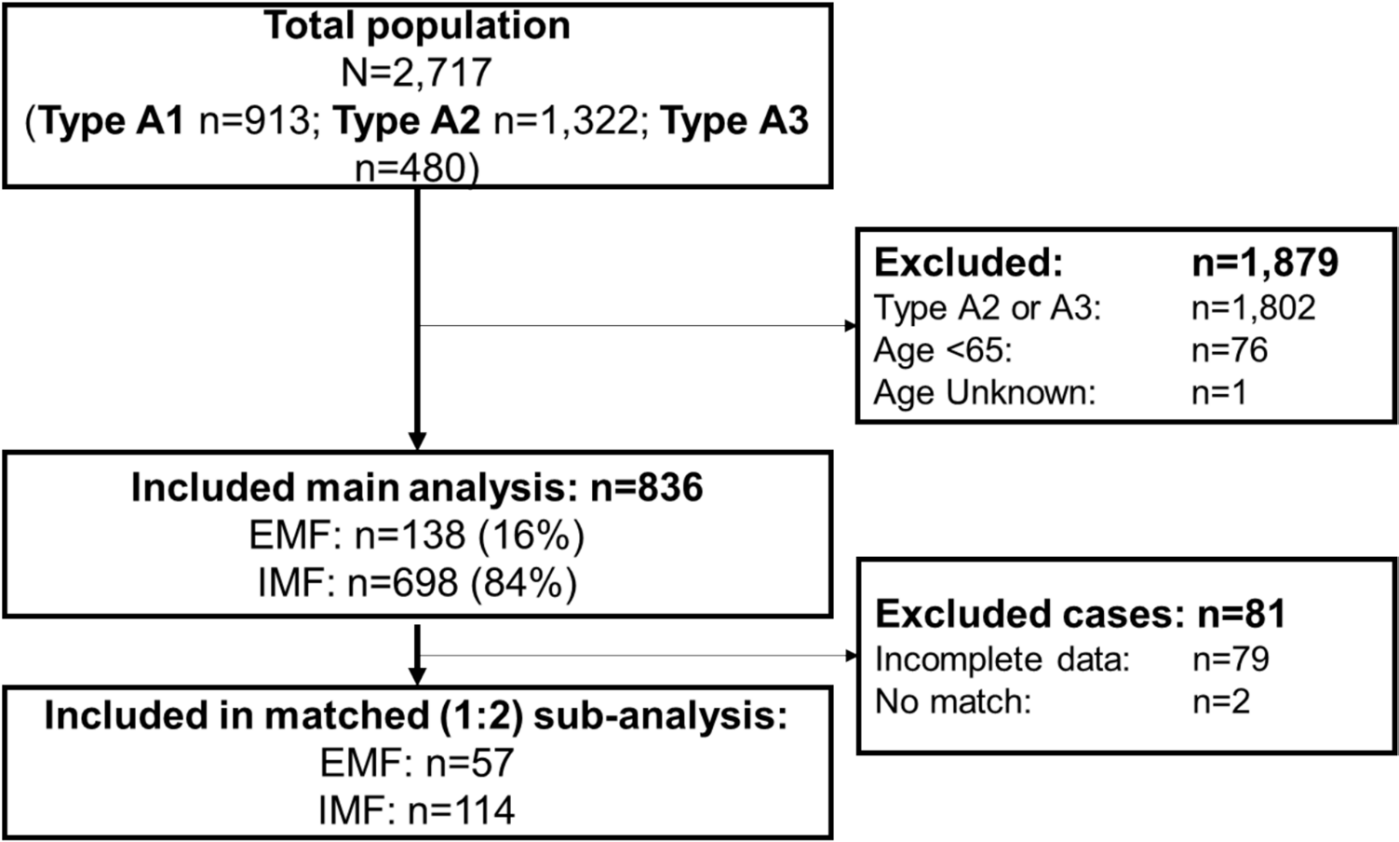
Flowchart of included and excluded patients

Out of a total population of 2,717 patients with trochanteric fractures, 913 patients were treated for a stable trochanteric femoral fracture in the participating hospitals (Fig. 1). Seventy-seven additional patients were excluded as they did not match the inclusion criteria (age ≥ 65). A total of 836 patients were included; 138 (16%) were treated with EMF and 698 (84%) with IMF (Table 1). These rates were similar during the three included years. The population had a median age of 84 (P_25_–P_75_ 76–89), and 71% was female. There were no significant differences in age, sex, ASA classification, PMS, or pre-fracture Katz ADL score between the EMF and IMF group. There was a significant difference in pre-fracture mobility, with a notably higher proportion of patients in the EMF group who were mobile with two supports or a frame (EMF 44% versus IMF 32%, p = 0.012). Another statistically significant difference was found for the pre-operative living status (p = 0.038). EMF patients were more likely to live unassisted at home (EMF 56% versus IMF 50%) and less likely to live in a care or nursing home (EMF 19% versus IMF 23%).

**Table 1:** Baseline characteristics of patients with a stable trochanter fracture treated with extramedullary or intramedullary fixation

Characteristic		EMF *n* = 138		IMF *n* = 698		
		n*		n*		*P*-value
Age		138	83 (76–89)	698	84 (76–89)	0.648
Female		138	95 (69%)	698	493 (71%)	0.683
Year injury	2018	138	52 (38%)	698	253 (36%)	0.802
	2019		44 (32%)		243 (35%)	
	2020		42 (30%)		202 (29%)	
Injured side	Right	138	73 (53%)	697	330 (47%)	0.227
ASA	1 or 2	136	51 (38%)	693	247 (36%)	0.696
	> 3		85 (63%)		446 (64%)	
Pre-fracture mobility	Independent mobility	131	59 (45%)	660	306 (46%)	**0.012**
	Mobile with 1 support		3 (2%)		30 (5%)	
	Mobile with 2 supports or frame		58 (44%)		210 (32%)	
	Mobile with support within own home		8 (6%)		100 (15%)	
	No functional mobility		3 (2%)		14 (2%)	
Pre-fracture Katz ADL score	0	134	75 (56%)	670	305 (46%)	0.083
	1		13 (10%)		61 (9%)	
	2		17 (13%)		71 (11%)	
	3		3 (2%)		48 (7%)	
	4		9 (7%)		57 (9%)	
	5		6 (5%)		63 (9%)	
	6		11 (8%)		65 (10%)	
Pre-fracture living status	Home, no supportive care	123	69 (56%)	683	340 (50%)	**0.049**
	Home, with supportive care		28 (23%)		148 (22%)	
	Care home		7 (7%)		57 (8%)	
	Nursing home		15 (12%)		104 (15%)	
	Rehabilitation institution		4 (3%)		7 (1%)	
	Other		0 (0%)		27 (4%)	

Data are shown as median (P_25_-P_75_) or as n (%). n*, number of patients for whom data were available. Bold values indicate statistical significance

*EMF* extramedullary fixation, *IMF* intramedullary fixation

### Operation and hospital stay

The median time to operation was 21 h (P_25_–P_75_ 14.9–27.6); Table 2). Median HLOS was similar for both groups (EMF 7 days (5–10) versus IMF 7 days (5–10), p = 0.717). During hospital stay EMF (55 patients, 40%) and (270 patients, 39%) IMF patients suffered a similar rate of complications (p = 0.849). The most common complications in both groups were anemia (19% versus 21%), delirium (11% versus 13%), urinary tract infection (7% versus 6%), and pneumonia (7% versus 5%). There were no significant differences between groups for all reported complications and in-hospital mortality.

**Table 2:** Outcomes for operation and initial hospital stay for extramedullary versus intramedullary fixation of stable trochanteric fractures

Outcome		EMF n = 138		IMF n = 698		
		n*		n*		P-value
Time to operation	Time (hours)	138	22 (17–27)	698	21 (14–28)	0.113
	< 24 h		84 (61%)		461 (66%)	0.402
	24–48 h		46 (33%)		193 (28%)	
	> 48 h		8 (46%)		44 (6%)	
Complication rate		138	55 (40%)	697	270 (39%)	0.796
Complication type	Anemia	138	26 (19%)	697	143 (21%)	0.717
	Cardiac decompensation	138	5 (4%)	697	22 (3%)	0.752
	Delirium	138	15 (11%)	697	88 (13%)	0.609
	Fall	138	2 (2%)	697	3 (< 1%)	0.150
	Infected wound	138	1 (1%)	697	1 (< 1%)	0.197
	Kidney failure	138	2 (1%)	697	8 (1%)	0.766
	Pneumonia	138	9 (7%)	697	32 (5%)	0.315
	Pressure ulcer	138	2 (1%)	697	12 (2%)	0.837
	Pulmonary embolism	138	1 (1%)	697	1 (< 1%)	0.202
	UTI	138	9 (7%)	697	41 (6%)	0.772
In-hospital mortality		138	6 (4%)	698	12 (2%)	0.052
HLOS (days)		132	7 (5–10)	685	7 (5–10)	0.717
Discharge location	Home, no supportive care	120	15 (13%)	680	53 (8%)	0.054
	Home with supportive care		12 (10%)		83 (12%)	
	Care home		7 (6%)		64 (10%)	
	Nursing home		18 (15%)		166 (24%)	
	Rehabilitation institution		64 (53%)		294 (43%)	
	Other		4 (3%)		20 (3%)	
Mobility at discharge	Independent mobility	132	1 (1%)	643	4 (1%)	** < 0.001**
	Mobile with 1 support		6 (5%)		22 (3%)	
	Mobile with 2 supports or frame		93 (71%)		295 (46%)	
	Mobile with support within own home		19 (14%)		181 (28%)	
	No functional mobility		13 (10%)		141 (22%)	

Data are shown as median (P_25_-P_75_) or as n (%). n*, number of patients for whom data were available. Bold values indicate statistical significance

*EMF* extramedullary fixation, *HLOS* hospital length-of-stay, *IMF* intramedullary fixation, *UTI* urinary tract infection

Despite differences before trauma, discharge location was not significantly different between groups. When comparing differences in living status pre-trauma and living status at discharge, no significant difference was found (p = 0.186, Online resource 1, Table S1) But similar to the pre-trauma situation, a larger proportion of EMF patients mobilized with two supports or a frame at discharge in comparison with IMF patients (EMF 71% versus IMF 46%) and IMF patients had a higher proportion of restricted mobility within their own homes (EMF 14% versus IMF 28%) or no functional mobility (EMF 10% versus IMF 22%). The proportion of patients with no functional mobility or only functional mobility within their own home increased more at discharge after surgery for the IMF group (EMF 9% increase versus IMF 28% increase).

### Three-month follow-up

Living status, mobility, and Katz-ADL score at three months were all similar between groups, despite differences in mobility at discharge (Table 3). In the EMF group, 29 (40.8%) patients were fully independent in ADL (Katz 0) versus 148 (43%) patients in the IMF group (p = 0.793). The total mortality rate was 16% in the EMF group versus 15% in the IMF group (p = 0.876). There was no statistically significant difference in reoperation rate between EMF (6 patients, 7%) and IMF (45 patients, 10%, p = 0.433). When comparing changes (from the pre-trauma situation to three months follow-up) in mobility, living status, and Katz-ADL score no significant differences between groups were found (Table 4). 43% of the EMF group and 46% of the IMF group returned to the previous level of mobility within three months (OR 0.9, 95% CI 0.5–1.5, p = 0.612). Specific changes in living status can be found in Online resource 1.

**Table 3:** Three-month outcomes and mortality for extramedullary versus intramedullary fixation of stable trochanteric fractures

		EMF n = 138		IMF n = 698		
Outcome		n*	n (%)	n*	n (%)	P-value
Living status at 3 months	Home, no supportive care	51	18 (35%)	323	157 (49%)	0.222
	Home with supportive care		16 (31%)		63 (20%)	
	Care home		2 (4%)		19 (6%)	
	Nursing home		9 (18%)		58 (18%)	
	Rehabilitation institution		6 (12%)		22 (7%)	
	Other		0 (0%)		4 (1%)	
Mobility at 3 months	Independent mobility	75	10 (13%)	362	51 (14%)	0.990
	Mobile with 1 support		8 (11%)		46 (13%)	
	Mobile with 2 supports or frame		42 (56%)		195 (54%)	
	Mobile with support within own home		9 (12%)		42 (11%)	
	No functional mobility		6 (8%)		28 (8%)	
Katz-ADL at 3 months	0	71	29 (41%)	343	147 (43%)	0.694
	1		13 (18%)		42 (12%)	
	2		10 (14%)		39 (11%)	
	3		3 (4%)		30 (9%)	
	4		4 (6%)		19 (6%)	
	5		8 (11%)		40 (12%)	
	6		4 (6%)		26 (8%)	
Reoperation < 3 months		89	6 (7%)	476	45 (10%)	0.546
Mortality at 3 months		96	15 (16%)	498	74 (15%)	0.876

Data are shown as median (P25-P75) or as n (%). n*, number of patients for whom data were available

*EMF* extramedullary fixation, *IMF* intramedullary fixation

**Table 4:** Changes in mobility, living situation, and KATZ-ADL score between pre-trauma and three-months after trauma; Crude analysis and Matched subgroup-analysis

		EMF n = 138		IMF n = 698			
Unmatched analysis		n*		n*		OR (95% CI)	P-value
Mobility	Decreased mobility	75	43 (57%)	350	189 (54%)	0.9 (9.5–1.5)	0.612
Living situation	From living at home to institution^*^	51	10 (20%)	312	48 (15%)	1.4 (0.6–2.9)	0.416
Katz-ADL	Decrease in 1 domain or more	70	27 (39%)	341	127 (37%)	1.0 (0.6–1.6)	0.892
Mortality		96	15 (16%)	498	74 (15%)	0.9 (0.5–1.7)	0.847

		EMF N = 57		IMF N = 114			
Matched complete case analysis^#^		n*		n*		OR (95% CI)	P-value
Mobility	Decreased mobility	43	20 (46%)	79	39 (49%)	1.2 (0.6–2.6)	0.615
Living situation	From living at home to institution^*^	43	8 (19%)	79	12 (15%)	0.8 (0.3–2.1)	0.649
Katz-ADL	Decrease in 1 domain or more	43	18 (41%)	79	28 (35%)	1.3 (0.6–2.8)	0.485
Mortality		57	14 (25%)	114	35 (30%)	0.7 (0.4–1.5)	0.403

Data are shown as n (%). n*, number of patients for whom data were available

^*^Return to home environment (with or without support) or return to any type of institution

^#^Patients matched 1:2 case:control on age, sex, pre-fracture mobility, and ASA classification

*EMF* extramedullary fixation, *IMF* intramedullary fixation

In the matched complete-case analysis 57 EMF patients were included and matched 1:2 to 114 IMF patients. After matched comparison, no significant differences were found in changes in mobility, living status, or Katz-ADL score (Table 4). For EMF 20/43 (46%) patients versus 39/79 (49%) IMF patients had decreased mobility after three months (OR 1.2, 95%CI 0.6–2.6, p = 0.615) and 8/43 (19%) EMF patients versus 12/79 (15%) IMF patients changed from community dwelling to institutionalized from baseline to three-months follow-up (OR 0.8, 95%CI 0.3–2.1, p = 0.649).

A sensitivity analysis stratified on pre-trauma living conditions (community-dwelling versus institutionalized) found similar results as the main analysis (Online Resource 2, Table S3-S5). With no differences between the groups in all three-month outcomes and only a significant difference between groups for mobility at discharge in favor of EMF.

## Discussion

This study showed that the vast majority of stable (AO type 31-A1) trochanteric fractures is treated with intramedullary fixation. No statistically significant differences were found in HLOS and in-hospital complications. At three months after trauma, no differences between groups were found in the reoperation rate, living status, mobility, and mortality when comparing EMF with IMF. Matched comparison of complete cases and sensitivity analysis in institutionalized or community-dwelling patients showed similar results after three months follow-up.

The main analysis of this study suggested a small indication for a faster recovery of patients’ mobility in the three months after surgery for the IMF group. Despite a higher proportion of IMF patients having no functional mobility or only functional mobility within the home environment at baseline/pre-trauma and at discharge, there was no statistically significant difference in mobility at the three-month follow-up. A similar effect was found in the subgroup analysis for both institutionalized and community-dwelling patients. However, due to loss to follow-up at three months, these findings must be interpreted with care. These findings might indicate that while EMF patients leave the hospital with better (or with a smaller decrease in) mobility, they make a slower functional recovery than IMF patients after three months. Although, when comparing changes in mobility before trauma and after three months, no significant differences were found in both the unmatched and matched analysis. Another recent registry study from Sweden, on both stable and unstable trochanteric fractures in independently living patients found no significant difference in return to independent living or mortality for stable trochanteric fractures [21]. Van der Sijp et al*.* did not specifically study mobility but found, in agreement with the current study’s results, no significant differences in Katz ADL score at three months and at one year follow-up [15]. Additionally, a previous randomized trial that combined both stable and unstable trochanteric fractures reported a significantly faster improvement of mobility (using the Parker mobility score) at three months for IMF in the A1 subgroup [22]. This trend continued up to one year follow-up. This might indicate that IMF has advantageous effects on mobilization of patients in the months after surgery, which may have beneficial effects on both fracture healing and quality of life.

A recent review comparing EMF and IMF for stable trochanteric fractures also found no meaningful differences in functional outcomes, complications, and surgical outcomes/operation characteristics [23]. It showed lower rates of reoperation for both fixation groups than the current study (EMF 3.1% versus IMF 3.6%) but found no significant difference between the groups, as well. The lower reoperation rate might be due to inclusion of, often more selective, and younger randomized trial populations in this study. As specific reasons for reoperation were poorly reported in the DHFA dataset, these could not be adequately compared. With respect to in-hospital outcomes and complications, the current study reported similar results to previous literature, with no significant differences in HLOS, in-hospital mortality, and complications at discharge [15, 22, 24, 25].

**Fig. 2:**
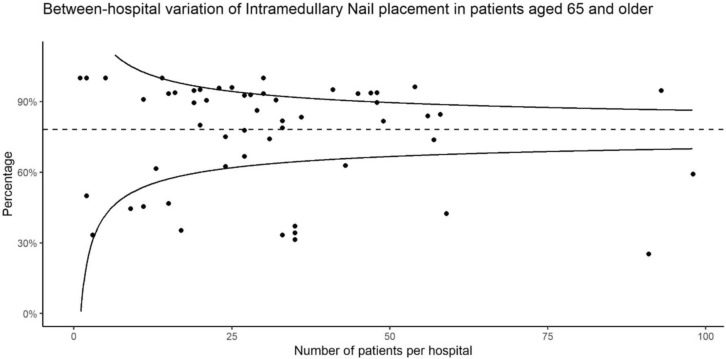
Between-hospital variation of intramedullary nail use for type 31-A1 trochanteric fractures in patients ≥ 65 years in 2018–2020 across the Netherlands. Each data point represents one Dutch hospital. This figure was provided by the Dutch Institute for Clinical Auditing and based on the complete national Dutch Hip Fracture Audit

The proportion of IMF versus EMF in this study was similar to data published for the full national coverage of the Dutch Hip Fracture Audit data in 2018–2019 (the current study also included data for the year 2020) [17]. This was also visualized in DHFA data for all Dutch hospitals, including the six centers studied in this analysis, in Fig. 2. This figure shows that the DHFA Indicator Taskforce is a representative sample of the national DHFA coverage and again confirms a low guideline adherence by Dutch (orthopedic) surgeons in the treatment of stable trochanteric fractures [7, 17]. International studies show that reasons for choosing a particular device are often not motivated by patient specific factors but by surgeon specific training or preferences [11, 16]. The difference in costs between both devices is significant and can range from around €300 to well over €2000 depending on the specific intramedullary device used, although specific costs per device are highly health system and also individual hospital dependent [26, 27]. If treatment of trochanteric fractures continues to shift towards only intramedullary nailing and EMF becomes a more obscure treatment, future generations of surgeons and surgical teams will lose the necessary training and experience. Future superiority of, the more expensive implant, IMF, will then become a self-fulfilling prophecy. As both devices are still used with relative frequency throughout the Netherlands, this might be a tipping point for a meaningful cost-effectiveness analysis. If extramedullary fixation proves to be more cost-effective, de-implementation studies and education aimed at increasing the use of EMF and decreasing the use of IMF for stable trochanteric fractures could potentially reverse the current trend.

## Limitations

Although the DHFA should include all (31-A1) hip fracture patients, due to the often retrospective completion of the DHFA follow-up using patient medical files, there is a risk of selection bias through asymmetry in missing data. Differences found in baseline characteristics indicate the possibility of indication bias, as patients in the EMF group had a statistically significant better mobility and living status at baseline. However, after sensitivity analysis, stratified on pre-fracture living status and matched complete case analysis, we found similar results after 3 month follow-up as in the main analysis. Due to the nature of the DHFA registration, we could not provide data for a longer follow-up period than three months. While this would not influence data concerning the initial hospital stay, outcomes like reoperation rate may increase further in the period after the three month follow-up. Due to the anonymized nature of the DHFA data, we could not compare the results of individual hospitals. Hospitals with lower usage of a specific device could have worse results. Data on specific causes for reoperation, surgical outcomes, specific nail subtypes or augmentation are not included in the DHFA and could therefore not be included in this analysis. Due to the limitations of the DHFA for the included years, data on interdisciplinary orthogeriatric treatment, an important factor in rehabilitation could not be included in this manuscript. However, orthogeriatric treatment is the standard of care in the Netherlands for older hip fracture patients and most of the included hospitals in this analysis operate a geriatric trauma unit [7].

## Conclusion

This study showed no meaningful differences between extramedullary and intramedullary fixation of stable (AO type 31-A1) trochanteric fractures during hospital stay and until three month follow-up. There were no significant differences between the groups for (deterioration of) mobility or living status after three months follow-up. Despite little differences in outcomes and extramedullary fixation being the treatment option of first choice by the Dutch Hip fracture guideline, intramedullary fixation was used in the vast majority of patients. Future efforts should focus more on cost-effectiveness analysis of both implant types.

## Supplementary Information

Below is the link to the electronic supplementary material.

Supplementary file1 (DOCX 17 KB)

Supplementary file2 (DOCX 30 KB)

## Data Availability

No datasets were generated or analysed during the current study.
