# Supplementary material for: Mobility and living status at discharge and after three-months for extramedullary versus intramedullary fixation of AO type 31-A1 trochanteric fractures; an analysis of Dutch hip fracture audit data
Source: Eur J Trauma Emerg Surg. 2025 Jan 10;51(1):6. doi: 10.1007/s00068-024-02749-8 (PMC11723844; doi:10.1007/s00068-024-02749-8)
Supplement: Supplementary file 1 — Supplementary file1 (DOCX 17 KB) [file 68_2024_2749_MOESM1_ESM.docx]

**Supplemental Materials 1: Changes in living status at discharge and follow-up**

**Table S1: Changes in living status at discharge compared with the pre-trauma situation for extramedullary versus intramedullary fixation of stable trochanteric fractures**

| **Living status** | | **EMF**  **n=138** | | **IMF**  **n=698** | |  |
| --- | --- | --- | --- | --- | --- | --- |
| **Pre-fracture** | **At discharge** | **n *** |  | **n*** |  | **P-value** |
| - | Discharge to pre-fracture location | 115 | 35 (30%) | 623 | 209 (34%) | 0.186 |
| No supportive care | Supportive care at home |  | 11 (10%) |  | 54 (9%) |  |
| No supportive care | Care home |  | 4 (4%) |  | 12 (2%) |  |
| No supportive care | Nursing home |  | 2 (2%) |  | 23 (4%) |  |
| No supportive care | Rehabilitation institution |  | 34 (30%) |  | 183 (29%) |  |
| Supportive care at home | Care home |  | 0 (0%) |  | 9 (1%) |  |
| Supportive care at home | Nursing home |  | 2 (2%) |  | 20 (3%) |  |
| Supportive care at home | Rehabilitation institution |  | 24 (21%) |  | 84 (14%) |  |
| Care home | Nursing home |  | 0 (0%) |  | 12 (2%) |  |
| Care home | Rehabilitation institution |  | 3 (3%) |  | 6 (1%) |  |
| Nursing home | Rehabilitation institution |  | 0 (0%) |  | 4 (1%) |  |
| Other or unknown | Other or unknown |  | 0 (0%) |  | 7 (1%) |  |

Data are shown as n (%). n*, number of patients for whom data were available.

EMF, extramedullary fixation; IMF, intramedullary fixation.

**Table S2: Changes in living status at three-month follow-up compared with pre-trauma for extramedullary versus intramedullary fixation of stable trochanteric fractures**

| **Living status** | | **EMF**  **n=138** | | **IMF**  **n=698** | |  |
| --- | --- | --- | --- | --- | --- | --- |
| **Pre-fracture** | **Three-month follow-up** | **n *** |  | **n*** |  | **P-value** |
| - | Living at pre-fracture location | 51 | 27 (53%) | 302 | 201 (67%) | 0.133 |
| No supportive care | Supportive care at home |  | 12 (24%) |  | 34 (11%) |  |
| No supportive care | Care home |  | 0 (0.0%) |  | 4 (1%) |  |
| No supportive care | Nursing home |  | 4 (8%) |  | 11 (4%) |  |
| No supportive care | Rehabilitation institution |  | 4 (8%) |  | 8 (3%) |  |
| Supportive care at home | No supportive care |  | 0 (0%) |  | 10 (3%) |  |
| Supportive care at home | Care home |  | 1 (2%) |  | 4 (1%) |  |
| Supportive care at home | Rehabilitation institution |  | 1 (2%) |  | 12 (4%) |  |
| Care home | Nursing home |  | 1 (2%) |  | 7 (2%) |  |
| Care home | Rehabilitation institution |  | 0 (0%) |  | 1 (0.3%) |  |
| Other or unknown | Other or unknown |  | 1 (2%) |  | 10 (3%) |  |

Data are shown as n (%). n*, number of patients for whom data were available.

EMF, extramedullary fixation; IMF, intramedullary fixation
