# Supplementary material for: Mobility and living status at discharge and after three-months for extramedullary versus intramedullary fixation of AO type 31-A1 trochanteric fractures; an analysis of Dutch hip fracture audit data
Source: Eur J Trauma Emerg Surg. 2025 Jan 10;51(1):6. doi: 10.1007/s00068-024-02749-8 (PMC11723844; doi:10.1007/s00068-024-02749-8)
Supplement: Supplementary file 2 — Supplementary file2 (DOCX 30 KB) [file 68_2024_2749_MOESM2_ESM.docx]

**Supplemental Materials 2: Subgroup analysis institutionalized and community-dwelling patients**

| **Characteristic** |  | **Institutionalized** | | | | | **Community dwelling** | | | | |
| --- | --- | --- | --- | --- | --- | --- | --- | --- | --- | --- | --- |
|  |  | **EMF**  **n=26** | | **IMF**  **n=168** | |  | **EMF**  **n=100** | | **IMF**  **n=497** | |  |
|  |  | **n*** |  | **n*** |  | **P-value** | **n*** |  | **n*** |  | **P-value** |
| Age |  | 26 | 89 (83-92) | 168 | 88 (82-92) | 0.581 | 100 | 81 (74-87) | 497 | 82 (74-89) | 0.407 |
| Female |  | 26 | 18 (69%) | 168 | 129 (77%) | 0.403 | 100 | 66 (66%) | 496 | 342 (69%) | 0.562 |
| Year injury | 2018 | 26 | 12 (46%) | 168 | 65 (39%) | 0.093 | 100 | 40 (40%) | 497 | 175 (35%) | 0.642 |
|  | 2019 |  | 13 (50%) |  | 66 (39%) |  |  | 31 (31%) |  | 172 (35%) |  |
|  | 2020 |  | 1 (4%) |  | 37 (22%) |  |  | 29 (29%) |  | 150 (30%) |  |
| Injured side | Right | 26 | 12 (46%) | 168 | 88 (52%) | 0.674^a^ | 100 | 54 (54%) | 496 | 222 (45%) | 0.100 |
| ASA | 1 or 2 | 25 | 3 (12%) | 166 | 33 (20%) | 0.424^a^ | 99 | 45 (46%) | 494 | 205 (42%) | 0.504 |
|  | >3 |  | 22 (88%) |  | 133 (80%) |  |  | 54 (55%) |  | 289 (59%) |  |
| Pre-fracture mobility | Independent mobility | 21 | 2 (10%) | 154 | 21 (14%) | 0.341 | 99 | 55 (56%) | 481 | 277 (58%) | 0.073 |
|  | Mobile with 1 support |  | 0 (0%) |  | 5 (3%) |  |  | 3 (3%) |  | 23 (5%) |  |
|  | Mobile with 2 supports |  | 15 (71%) |  | 74 (48%) |  |  | 36 (36%) |  | 130 (27%) |  |
|  | Mobile with support within own home |  | 3 (14%) |  | 46 (30%) |  |  | 3 (3%) |  | 47 (10%) |  |
|  | No functional mobility |  | 1 (5%) |  | 8 (5%) |  |  | 2 (2%) |  | 4 (1%) |  |
| Parker mobility score |  | 24 | 2 (2-4) | 140 | 4 (2-6) | 0.122 | 89 | 9 (6-9) | 448 | 9 (6-9) | 0.152 |
| Pre-fracture KATZ ADL | Independent (KATZ 0) | 23 | 7 (30%) | 157 | 12 (8%) | **0.004^a^** | 100 | 68 (68%) | 483 | 285 (59%) | 0.115 |
|  | Dependent (KATZ>0) |  | 16 (70%) |  | 145 (92%) |  |  | 32 (32%) |  | 198 (41%) |  |

**Table S3: Baseline characteristics of institutionalized and community-dwelling patients with a stable (A1) trochanteric fracture treated with extramedullary or intramedullary fixation**

Data are shown as median (P25-P75) or as n (%). n*, number of patients for whom data were available. Bold values indicate statistical significance.

EMF, extramedullary fixation; IMF, intramedullary fixation.

| **Outcome** |  | **Institutionalized** | | | | | **Community-dwelling** | | | | |
| --- | --- | --- | --- | --- | --- | --- | --- | --- | --- | --- | --- |
|  |  | **EMF**  **n=26** | | **IMF**  **n=168** | |  | **EMF**  **n=100** | | **IMF**  **n=497** | |  |
|  |  | **n*** |  | **n*** |  | **P-value** | **n*** |  | **n*** |  | **P-value** |
| Time to operation | Time (hours) | 26 | 23 (17-29) | 167 | 21 (13-29) | 0.393 | 100 | 22 (17-25) | 496 | 20 (15-27) | 0.333 |
|  | <24 hours | 26 | 14 (53%) | 167 | 101 (61%) | 0.090 | 100 | 65 (65%) | 496 | 336 (68%) | 0.814 |
|  | 24-48 hours |  | 12 (46%) |  | 49 (29%) |  |  | 28 (28%) |  | 132 (27%) |  |
|  | >48 hours |  | 0 |  | 17 (10%) |  |  | 7 (7%) |  | 28 (6%) |  |
| Complication rate |  | 26 | 15 (58%) | 168 | 69 (41%) | 0.137^a^ | 100 | 35 (35%) | 496 | 190 (38%) | 0.573 |
| Complication type | Anemia | 26 | 8 (31%) | 168 | 37 (22%) | 0.326^a^ | 100 | 16 (16%) | 496 | 100 (20%) | 0.406 |
|  | Cardiac decompensation | 26 | 2 (8%) | 168 | 8 (5%) | 0.626^a^ | 100 | 1 (1%) | 496 | 14 (3%) | 0.486 |
|  | Delirium | 26 | 5 (20%) | 168 | 20 (12%) | 0.342^a^ | 100 | 9 (9%) | 496 | 63 (13%) | 0.400 |
|  | Fall | 26 | 1 (4%) | 168 | 1 (1%) | 0.251^a^ | 100 | 1 (1%) | 496 | 2 (<1%) | 0.424 |
|  | Infected wound | 26 | 0 | 168 | 0 | N.A. | 100 | 1 (1%) | 496 | 1 (<1%) | 0.308 |
|  | Kidney failure | 26 | 0 | 168 | 2 (1%) | 1.000^a^ | 100 | 2 (2%) | 496 | 6 (1%) | 0.627 |
|  | Pneumonia | 26 | 2 (8%) | 168 | 11 (7%) | 0.687^a^ | 100 | 6 (6%) | 496 | 21 (4%) | 0.431 |
|  | Pressure ulcer | 26 | 0 | 168 | 3 (2%) | 1.000^a^ | 100 | 2 (2%) | 496 | 9 (2%) | 1.000 |
|  | Pulmonary embolism | 26 | 0 | 168 | 0 | N.A. | 100 | 1 (1%) | 496 | 1 (<1%) | 0.308 |
|  | UTI | 26 | 3 (12%) | 168 | 6 (4%) | 0.104^a^ | 100 | 5 (5%) | 496 | 34 (7%) | 0.658 |
| In-hospital mortality |  | 26 | 5 (20%) | 168 | 4 (2%) | **0.003^a^** | 100 | 0 | 497 | 8 (2%) | 0.364 |
| HLOS (days) |  | 21 | 4 (4-6) | 163 | 5 (4-7) | 0.429 | 100 | 7 (5-12) | 489 | 8 (6-11) | 0.375 |
| Discharge to | Institution | 21 | 21 (100%) | 163 | 163 (100%) | 1.000^a^ | 98 | 71 (72%) | 485 | 349 (72%) | 1.000 |
| Mobility at discharge | Independent mobility | 21 | 0 | 153 | 2 (1%) | **0.017** | 100 | 1 (1%) | 465 | 2 (<1%) | **0.006** |
|  | Mobile with 1 support |  | 0 |  | 3 (2%) |  |  | 6 (6%) |  | 18 (4%) |  |
|  | Mobile with 2 supports or frame |  | 12 (57%) |  | 34 (22%) |  |  | 72 (72%) |  | 255 (55%) |  |
|  | Mobile within home |  | 3 (14%) |  | 48 (31%) |  |  | 15 (15%) |  | 125 (27%) |  |
|  | No functional mobility |  | 6 (29%) |  | 66 (43%) |  |  | 6 (6%) |  | 65 (14%) |  |

**Table S4: Outcomes for operation and initial hospital stay for institutionalized and community dwelling patients with stable (A1) trochanteric fractures**

Data are shown as median (P_25_-P_75_) or as n (%). n*, number of patients for whom data were available. Bold values indicate statistical significance. EMF, extramedullary fixation; HLOS, hospital length-of-stay; IMF, intramedullary fixation; UTI, urinary tract infection.

**Table S5: Subgroup analysis of the three-month outcomes of institutionalized and community-dwelling patients comparing extramedullary versus intramedullary fixation**

| **Outcome** |  | **Institutionalized** | | | | | **Community-dwelling** | | | | |
| --- | --- | --- | --- | --- | --- | --- | --- | --- | --- | --- | --- |
|  |  | **EMF**  **n=26** | | **IMF**  **n=168** | |  | **EMF**  **n=100** | | **IMF**  **n=489** | |  |
|  |  | **n*** |  | **n*** |  | **P-value** | **n*** |  | **n*** |  | **P-value** |
| Reoperation <3 months |  | 12 | 1 (8%) | 102 | 12 (12%) | 1.000^a^ | 68 | 5 (7%) | 358 | 33 (9%) | 0.817 |
| Living status at 3 months | Home, no supportive care | 7 | 0 | 55 | 2 (4%) | 0.808 | 44 | 18 (41%) | 256 | 154 (60%) | 0.145 |
|  | Home with supportive care |  | 0 |  | 2 (4%) |  |  | 16 (36%) |  | 61 (24%) |  |
|  | Care home |  | 1 (14%) |  | 6 (11%) |  |  | 1 (2%) |  | 9 (4%) |  |
|  | Nursing home |  | 5 (71%) |  | 41 (75%) |  |  | 4 (9%) |  | 12 (5%) |  |
|  | Rehabilitation institution |  | 1 (14%) |  | 2 (4%) |  |  | 5 (11%) |  | 20 (8%) |  |
|  | Other |  | 0 |  | 2 (4%) |  |  | 0 |  | 0 |  |
| Mobility at 3 months | Independent mobility | 9 | 1 (11%) | 62 | 1 (2%) | 0.183 | 61 | 8 (13%) | 465 | 50 (18%) | 0.621 |
|  | Mobile with 1 support |  | 0 |  | 2 (3%) |  |  | 8 (13%) |  | 43 (15%) |  |
|  | Mobile with 2 supports |  | 3 (33%) |  | 34 (55%) |  |  | 36 (59%) |  | 154 (54%) |  |
|  | Mobile within own home |  | 5 (56%) |  | 18 (29%) |  |  | 3 (5%) |  | 22 (8%) |  |
|  | No functional mobility |  | 0 |  | 7 (11%) |  |  | 6 (10%) |  | 17 (6%) |  |
| Katz-ADL at 3 months | Independent (Katz 0) | 9 | 0 | 62 | 2 (3%) | 1.000^a^ | 57 | 28 (49%) | 270 | 142 (53%) | 0.664 |
|  | Dependent (Katz>0) |  | 9 (100%) |  | 60 (97%) |  |  | 29 (51%) |  | 128 (47%) |  |
| Mortality at 3 months |  | 17 | 6 (35%) | 107 | 25 (23%) | 0.365^a^ | 71 | 6 (8.5%) | 374 | 47 (13%) | 0.425 |

Data are shown as median (P25-P75) or as n (%). n*, number of patients for whom data were available.

EMF, extramedullary fixation; IMF, intramedullary fixation.
